# From IgG Fusion Proteins to Engineered-Specific Human Regulatory T Cells: A Life of Tolerance

**DOI:** 10.3389/fimmu.2017.01576

**Published:** 2017-11-13

**Authors:** David W. Scott

**Affiliations:** ^1^Department of Medicine, Uniformed Services University, Bethesda, MD, United States

**Keywords:** regulatory T cells, chimeric antigen receptor, engineered T cells, hemophilia A, multiple sclerosis, B-cell receptor, single-chain variable fragment

## Abstract

Recent efforts have concentrated on approaches to expand and “specify” human regulatory T cells (Tregs) and to apply them to modulate adverse immune responses in autoimmunity and hemophilia. We have used retroviral transduction of specific T-cell receptor, single chain Fv, or antigen domains in Tregs to achieve this goal. Each of these approaches have advantages and disadvantages. Results with these engineered T cells and evolution of the research developments and paths that led to the development of specific regulatory approaches for tolerance are summarized.

## Introduction

Self-non-self discrimination, i.e., immunologic tolerance, is a hallmark of the immune system. Implicit in this paradigm is specificity. Understanding how the immune system learns what is self can be demonstrated by a conversation from Sir Arthur Conan Doyle’s short story “Silver Blaze” about a murder that took place in the stable of this prize racehorse:

“Is there any other point to which you wish to direct my attention?” asked Dr. Watson.
“To the curious incident of the dog in the night time!”
“But the dog did nothing in the night time.”
“That,” remarked Sherlock Holmes, “was the curious incident.” (1)

As insightful as ever, the master detective realized that the watchdog in the stable recognized the culprit as “familiar” and thus did not respond. The watchdogs of the immune system, the T and B lymphocytes, also must learn what self (familiar) is and what is not (foreign) in order to provide specific responses to potential dangers. Immunologic tolerance must be learned (2, 3). This property of the immune system has driven research in my lab for decades, most recently in the area of specific regulatory T cells (Tregs). In this review, I will summarize the research that led to the development of specific Tregs to induce tolerance and reverse adverse immune responses.

Much of the early work was pioneered by the late Weigle and colleagues (4–6) with IgG as a tolerogen and extended by seminal studies from Yves Borel, who used IgG fusions as tolerogens (7, 8), the latter being shown to depend on the presence of the IgG Fc fragment (9, 10). Later, we used gene therapy of B cells expressing fusions of antigens with an IgG heavy chain to be highly tolerogenic in several systems (11–14) and showed that this approach was dependent on Tregs for both its induction and maintenance (15–17). Indeed, recent development of Fc fusions of clotting factors like Factor VIII (FVIII) and FIX, designed for a longer half-life *in vivo* (18–20), have turned out to be tolerogenic and to induce Tregs (21, 22), as discussed below. This is supported by anecdotal cases in hemophilia A patients that suggest that FVIII-Fc is potentially tolerogenic (23–25), which is leading to a more highly powered clinical trial (26). The reason that Fc fusions are tolerogenic is not precisely known, but may involve regulatory epitopes in the constant region (27, 28) that turn on Tregs, and/or inhibitory Fc receptors (29).

In this review, we will summarize the evolution of the research paths that led to the development of specific Treg approaches for tolerance. We have concentrated recently on efforts to expand and “specify” Tregs (30, 31) and apply them to modulate adverse immune responses in autoimmunity and hemophilia.

### Hemophilia A

Hemophilia A is an X-linked bleeding disorder caused by mutations in the FVIII (*F8*) gene. This gene encodes a 250 kDa protein, FVIII, which is a critical component of the blood coagulation cascade. Severe hemophilia A results from major deletions or inversions in the *F8* gene, such that these individual have less than 1% FVIII activity; mild hemophilia can occur with missense mutations, for example, that also lead to significantly reduced clotting efficacy. These disorders can be treated with recombinant or plasma-derived FVIII replacement therapy, either prophylactically or on demand. Unfortunately, a large subset of those receiving replacement FVIII develop an antidrug antibody response because they never developed tolerance to this human protein (unlike the dog in the nighttime!) In the hematology community, these antibodies are referred to as “inhibitors” because they can inhibit or neutralize the therapeutic function of FVIII, rendering this life-saving treatment ineffective. Inhibitor formation requires CD4^+^ T cell help as evidenced originally in HIV-infected patients with inhibitors whose titers dropped when their T-cell levels diminished, but whose antibodies returned upon multi-drug therapy (32, 33). Further studies in a murine model (FVIII knockouts) verified this T-cell dependence (34, 35). Most of the inhibitory antibodies are directed at the A2 and C2 domains of the FVIII protein, which are critical for binding to partners in the cascade.

For several decades, the standard treatment in patients that develop inhibitors is repeated, high-dose FVIII therapy to reduce or eliminate titers, a process referred to clinically as “immune tolerance induction.” This is an expensive process and does not work for all inhibitor cases, being successful primarily in patients with low-titered antibodies. Thus, we have targeted the A2 and C2 domains of the FVIII protein in our approaches for inducing tolerance to FVIII (13, 22). This would be important to achieve in inhibitor positive patients or to prevent inhibitor responses, in the first place, which is of clinical importance.

## Fc Fusions in Hemophilia and Other Disease Models

As noted above, IgG carriers have been shown to be highly tolerogenic. In part, this may reflect their long half-life in the circulation and even in tissues. In addition, binding to Fc receptors on B cells can deliver a negative signal that aborts full signaling (36). Teleologically, it is important that the immune system be tolerant of its own products. Immunoglobulins express an enormous range of specific receptors (idiotypes) that must be tolerated as their numbers increase and diversify during an immune response. Based on the hypothesis that IgG was a highly tolerogenic carrier, we devised a strategy to express a variety antigens in frame on an IgG heavy chain scaffold. Recombinant expression of these fusion proteins was predicted to be tolerogenic, and indeed they were (22, 37). We also reasoned that retroviral expression in B cells in which the fusion heavy chain would be assembled with endogenous light chains would lead to secretion of hybrid molecules into the circulation to tolerize the autologous host. Indeed, this also occurred (11). However, this was not due to the secreted product, but rather by B-cell tolerogenic presentation (38), confirming the work of Eynon and Parker (39) and Fuchs and Matzinger (40). Importantly, we found that B-cell expression of MHC class II and B7, but not Fc receptors on the transduced B cells was required (41–43), and that the IgG scaffold enhanced the tolerogenicity of these cells (44). Further data suggested that IgG may contain tolerogenic epitopes (27).

Over the next decade, we utilized this system to induce tolerance to a variety of antigens in multiple autoimmune disease models (uveitis, EAE, diabetes, arthritis) and in hemophilia A (12, 13, 15, 45–48). In many of these studies, a role for Tregs was suggested or demonstrated for the induction or maintenance of tolerance (16, 47). Thus, we embarked on an effort to develop a platform for Treg-based tolerance protocol, focusing on two different diseases, hemophilia and multiple sclerosis (MS). In the former, an adverse (T-cell dependent) antibody response blocks effective therapy, whereas in the latter, T-cell-mediated pathology in the central nervous system is the target.

## Rationale for Designing Specific Tregs

Polyclonal human Tregs have been proposed to treat autoimmune diseases and transplant rejection, as well as to suppress undesirable immune responses to bio-therapeutics such as recombinant or plasma-derived FVIII. Several of these are already in clinical trials (49–51). While these appear to be safe, they are polyclonal T cells that include a diverse repertoire of specificities and large numbers of polyclonal Tregs are needed. Thus, there is the possibility that non-specific immunosuppression and viral reactivation could occur (52). Moreover, the frequency of relevant specific Tregs is quite low in a normal repertoire. One could attempt to enrich and expand Tregs using antigen and/or tetramers in the presence of antigen-presenting cells (APC) and IL-2, as long as they do not revert to an effector pathogenic phenotype.

We elected instead to render human Tregs specific, based on chimeric antigen receptor (CAR) therapy for cancer (53–55), and to maintain their functional properties during expansion with a novel approach (56). Hence, we engineered specificity into polyclonal Tregs *via* retroviral transduction of specific T-cell receptors (TCR) or CARs [single-chain variable fragment (scFv)], or even antigen [B-cell antibody receptor (BAR)].

## Four Flavors of Specific Tregs

### TCR-Transduced CD4 T Cells

Inspired by the success of engineered cytotoxic CAR T cells in blood cancers (55), our goal was to apply this approach to directly create large numbers of specific Tregs with engineered receptors. As noted above, based on our experience with retroviral transduction of Fc fusions into activated B cells, we had established a role for Tregs in the tolerance so induced. The buffy coat fractions in all of the experiments to be described below were from peripheral blood mononuclear cells (PBMC) from healthy normal adult donors from the American Red Cross or the NIH Blood Bank. CD4 fractions were isolated by magnetic cell enrichment, then labeled, and sorted based upon the following cell surface markers: naïve CD4 effector T cells were CD4^+^, CD25^−^ CD127^+^, and CD45RA^+^ and Tregs were CD4^+^, CD25^high^, CD127^low^ (and the latter were Foxp3 and Helios positive, reflecting their status as “natural” Tregs).

**Figure 1:**
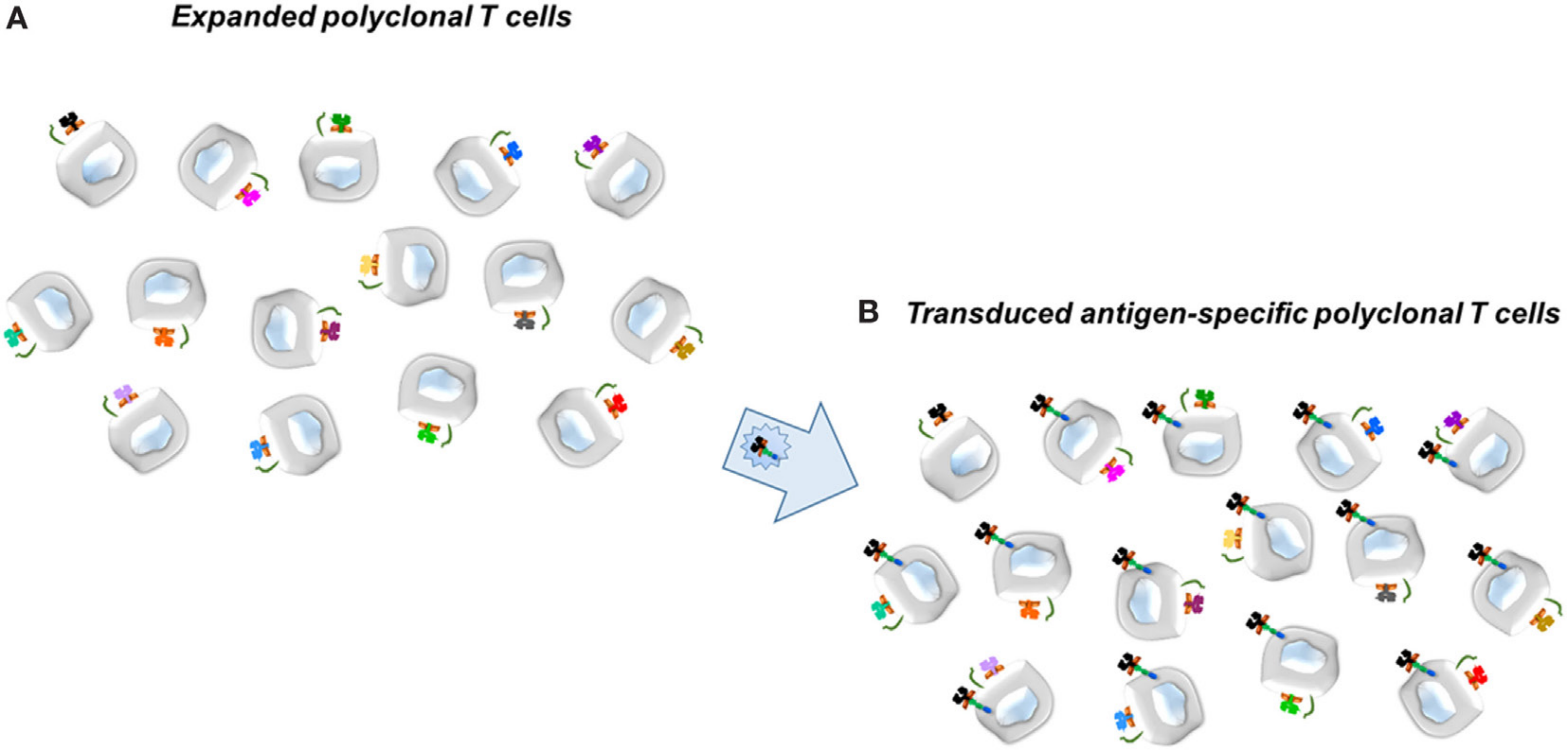
Scheme for transduction of Factor VIII-specific (T-cell) receptors into expanded human T cells, either CD4^+^ effector or CD25^hi^ regulatory T cells. **(A)** Expanded polyclonal T cells. **(B)** Transduced antigen-specific T cells. Retroviral vector to express the T-cell receptors, for example, is shown in the large arrow.

In collaboration with Dr. Kate Pratt, who had obtained multiple clones of FVIII-specific T effectors from patients with hemophilia A, we determined the TCR variable (V) region genes from two of these clones, termed 17195 and 171911. In the first iteration for specific CD4 effectors and Tregs, retroviral vectors were engineered to express the 17195 or 171911 TCR variable regions in polyclonal T cells activated initially with anti-human CD3. The transduced T cells were expanded as described by Kim et al. (30) with irradiated PBMC’s as APC. Notably, Tregs were expanded but their cultures also contained random oligonucleotides (ODNs), which Kim et al. (56) had shown serve to maintain Treg properties (Foxp3 and Helios). Figure 1 illustrates the principle.

With this approach, we obtained large numbers of expanded FVIII-specific T cells expressing the 17195 or 171911 TCRs that we demonstrated were highly reactive to the FVIII peptides, albeit with different affinities based on the affinity of the initial clones (57). The transduced T effectors proliferated and produced cytokines in response to the FVIII peptide (pC2, 2191–2210) on appropriate DR1 APCs just as effectively as anti-CD3 stimulation of the donors; moreover, specific antigen led to an expansion of the cells expressing the TCR as evidenced by tetramer binding (30). Transduced and expanded Tregs also responded to peptide and displayed increased levels of Foxp3, Helios, GARP, and LAP, typical of activated Tregs, but did not produce significant levels of IL-2 and interferon gamma (IFNγ). Thus, these cells looked like and smelled like human Tregs. We next tested whether they could suppress a FVIII-specific response and found that proliferation of FVIII-specific effector T cells was suppressed even when the effector cells were cultured at an 8:1 ratio to Tregs (30).

As noted above, the antibody response to FVIII in hemophilia A patients is a major hindrance to effective therapy for bleeding. Therefore, we have tested the effect of engineered FVIII-specific human Tregs on an *in vitro* recall antibody response to FVIII in humanized (DR1) hemophilic knockout mice, using the approach of Hausl et al. (58). Despite being a xenogeneic system, the engineered Tregs were able to suppress the recall antibody response to FVIII (30). Interestingly, although the engineered TCR recognizes a single peptide in a large protein, the antibody response to other major epitopes of FVIII was also suppressed. This indicates that bystander suppression of other T (and B) cells had occurred *in vitro*. Subsequently, we demonstrated that this could also occur *in vivo* so it was not due to a culture artifact (31). Thus, we have engineered specificity into expanding human Tregs and shown that they can suppress the antibody response to FVIII effectively.

### scFv Transduced CD4 T Cells

**Figure 2:**
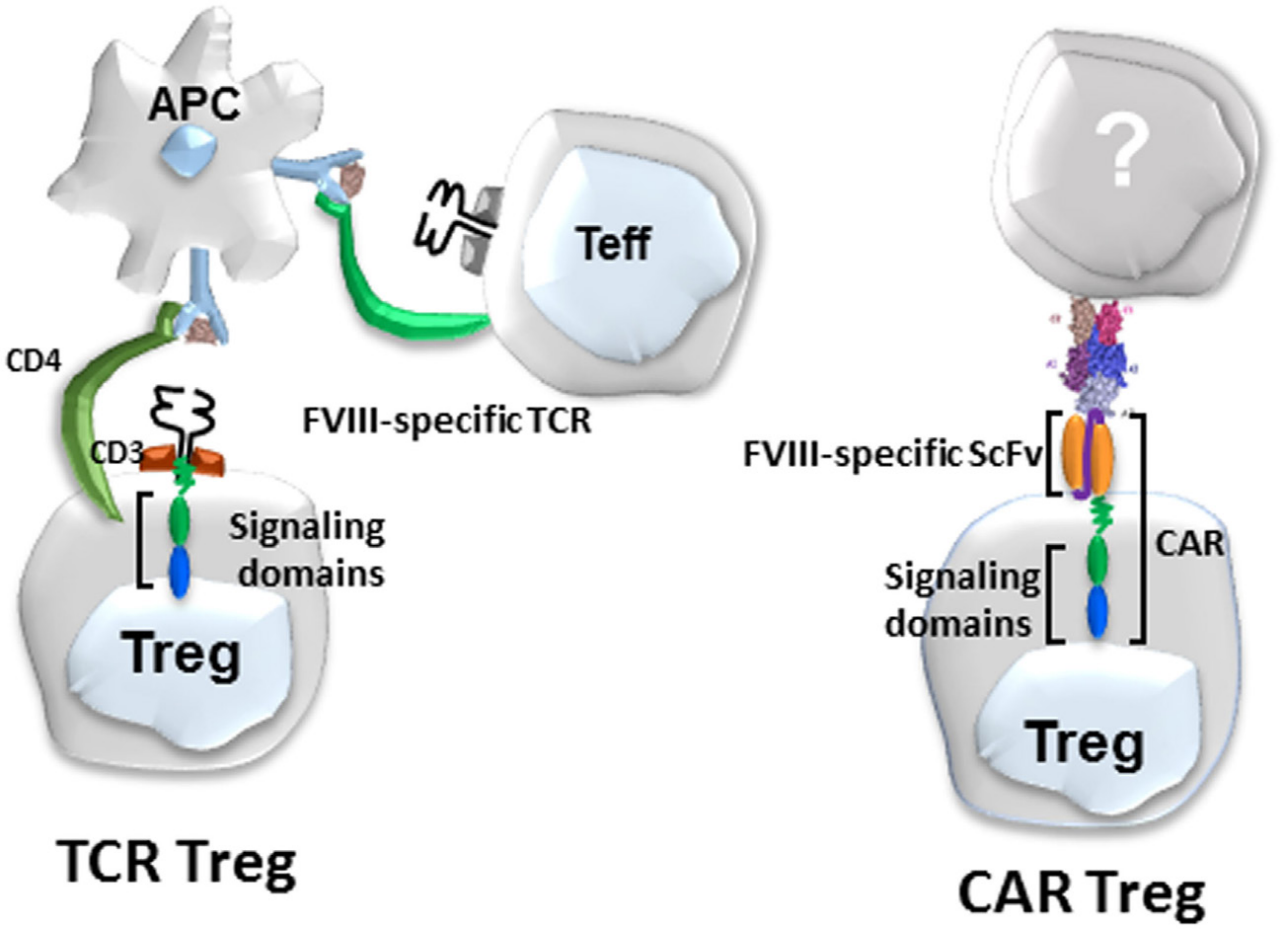
Diagram of Factor VIII (FVIII)-specific T-cell receptors (TCR)-transduced (left) and single-chain variable fragment (scFv)-transduced human regulatory T cells (Tregs) (right).

While these TCR-transduced Tregs were highly effective, they are MHC class II restricted, thus limiting their eventual utility to patients sharing the same MHC globally. Therefore, in collaboration with Anja Naumann Schmidt and Christoph Königs in Frankfurt, we developed a second approach to engineer specificity, namely a scFv, as shown in Figure 2. Dr. Schmidt used phage display to obtain a number of single chain antibodies that reacted with different domains of FVIII (59, 60). One of these, called ANS8, recognized the A2 domain of FVIII. This scFv was incorporated into our retroviral vector and used to transduce both CD4 effectors and Tregs. These scFv transduced cells recognized free FVIII but responded to membrane or plate bound FVIII more effectively (31), presumably reflecting the exposure of the A2 domain under these conditions.

ANS8 CAR human Tregs were generated and tested under the same conditions as the 17195 TCR Tregs. These Tregs also suppress the proliferation of FVIII-specific T effector cells, but most importantly suppressed the antibody response to FVIII both *in vitro* and *in vivo* (31). Notably, both the ANS8 CAR-transduced Tregs and 17195 (TCR)-transduced Tregs were effective in these assays at effector: target ratios with effector cells in excess (31). Suppression of the antibody response by these human Tregs *in vivo* lasted up to 8 weeks. When these mice were boosted with FVIII at 8 weeks post immunization, suppression was lost presumably because the human cells were rejected by the immunocompetent murine hosts. Nevertheless, these data demonstrate that both CAR- and TCR-transduced specific Tregs that recognize different B-cell and T-cell domains of FVIII can be suppressive against multiple epitopes of this large immunogenic protein. Despite this bystander effect, the response to an unrelated antigen (TNP-sheep RBC) was not affected. Thus, suppression in this model is specific.

### “BAR” Expressing CD4 Tregs and Cytotoxic T Cells

**Figure 3:**
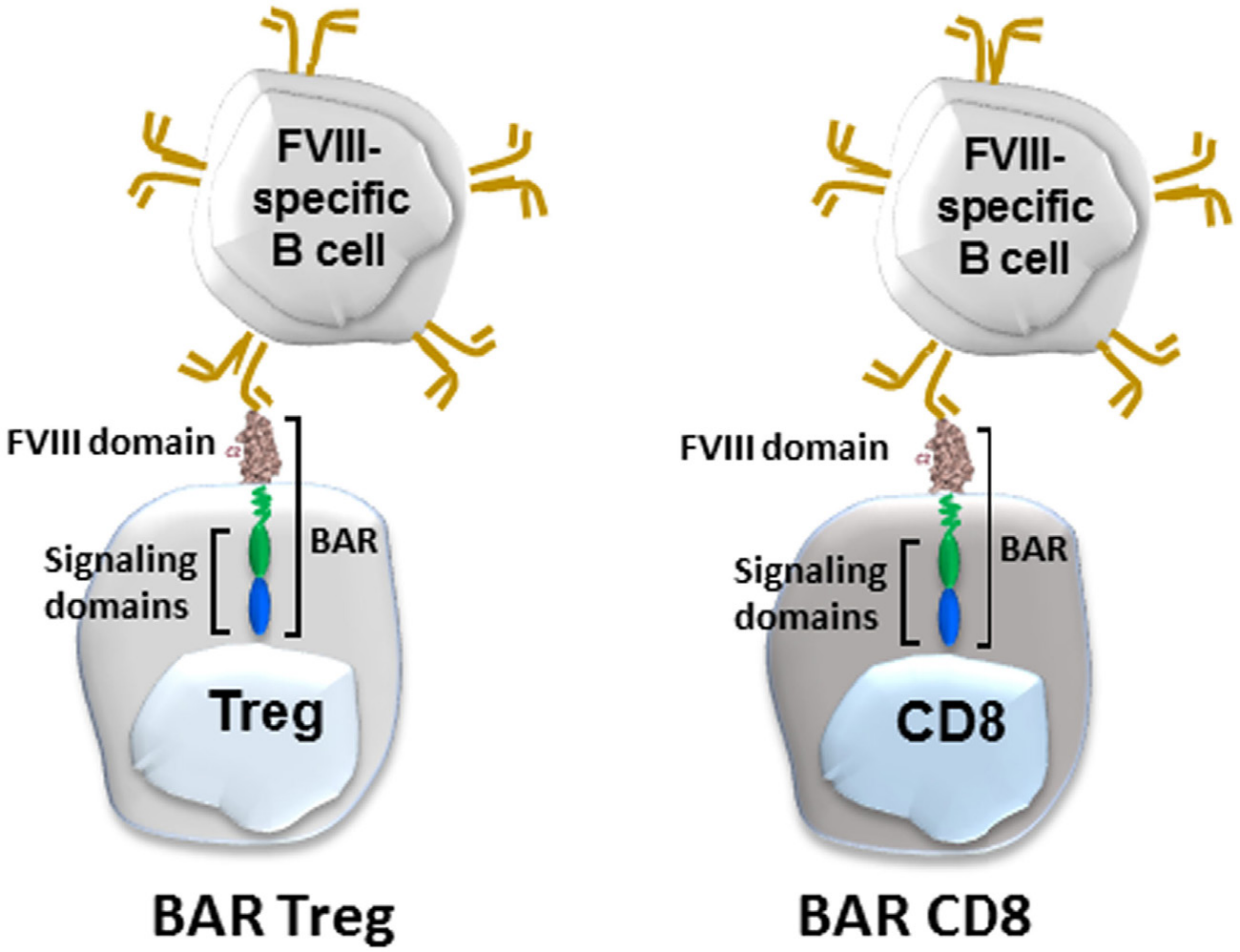
Diagram of BAR-transduced regulatory T cells (Tregs) (left) and BAR-transduced cytotoxic CD8 T cells (right). BAR stands for B-cell antibody receptor, which in this case is a Factor VIII (FVIII) domain. The BCR (gold) binds the antigen on the Treg or cytotoxic T cells *via* its variable regions, which signals the T cell.

We recently applied the principle of engineered cytotoxic CAR T cells to directly target FVIII-specific B cells. In lieu of a chimeric antibody, we engineered immunodominant B-cell domains of FVIII into both expanded cytotoxic CD8 and regulatory CD4 T cells (Figure 3). The principle hypothesis was that FVIII-specific B cells possess IgM and IgD receptors that recognize FVIII conformational epitopes. When they would encounter engineered cytotoxic T cells, for example, they would bind these epitopes to form a synapse and would receive a putative negative signal from these cytotoxic cells. This was recently demonstrated by Ellebrecht et al. (61), who used engineered cytotoxic T cells expressing a major skin target (desmoglein 3) in pemphigus vulgaris, a devastating skin disease. They showed that human cytotoxic T cells expressing desmoglein 3 could kill B-cell hybridomas specific for desmoglein. To apply this for hemophilia, we engineered immunodominant C2 or A2 domains (that are the major targets of inhibitory antibodies to FVIII into both human and mouse cytotoxic cells). These BAR expressing cytotoxic T cells were able to kill C2- and A2-specific hybridomas *in vitro* and *in vivo*. Moreover, their specificity for FVIII-specific B cells was formally demonstrated in two additional assays: elimination of naïve B cells stimulated with polyclonal B-cell activator, LPS, and inhibition of the antibody response to FVIII *in vivo*. Because they are domain-specific and did not display a bystander effect, both C2- and A2-BARs were needed to eliminate the response to full-length FVIII (62).

What about BAR Tregs? Theoretically, BAR-expressing Tregs could also interact with specific B cells, but we did not know whether they could directly or indirectly inhibit the B-cell response to FVIII. These data demonstrated that injections of human BAR Tregs into hemophilic mice did indeed prophylactically prevent the antibody response to FVIII (63). To examine the mechanism of this inhibition, we purified B and T cells from BAR tolerized and control mice and then performed classic mixing experiments. These results suggested that B cells may be directly targeted by BAR Tregs since they could not be “helped” by control non-tolerant T cells (64). Whether this is due to anergy or cytotoxicity of targeted B cells is under investigation.

## Discussion

Specific tolerance induction to treat a variety of adverse immune reactions is preferable to non-specific immune suppression. We have focused on the use of engineered specific Tregs and cytotoxic T cells and have developed four different approaches for applications to treat adverse immune responses in both monogenic diseases, like hemophilia (30, 31), as well as in autoimmunity. The notion of engineering specificity into T cells was pioneered by Eshhar (54, 65) and colleagues with an approach that he termed “T-bodies.” Subsequent application of engineered cytotoxic T cells in the treatment of hematologic cancers has revolutionized therapy for those diseases (55, 66). Recently, several others have engineered T-Regs targeting different antigens (67–69). The most analogous to our studies are those of MacDonald et al. (68), who utilized a single chain Fv that targeted the HLA class I antigen, A2. We have used retroviral expression in human T-Regs of specific TCRs and an scFv that recognize FVIII T- and B-cell epitopes, respectively, for hemophilia, as well as antigen domains that would be recognized by B cells, all of which were functionally stable and competent to suppress FVIII responses *in vitro* and *in vivo*. In addition, we have extended this approach with a myelin basic protein-specific TCR to suppress autoimmune responses in a model for MS (70).

The mechanism(s) of suppression are not fully understood. Recent data suggest that signals from IL-2 derived from effector cells “turn on” a program of suppression by the engineered Tregs, and that this leads to a bystander effect in the local milieu. Further characterization of the mediators is in progress.

Determination of which kind of engineered Tregs will be most applicable will depend in part on the target antigen(s) and the disease entity and effector targets. The process described herein is a personalized medicine that could be limited to autologous donors. Given this limitation, as well as HLA restriction and the possibility that Tregs may be defective in certain diseases (71, 72), we envision that a generic/universally applicable population of Tregs can be prepared in which CRISPR/Cas9 technology can be used to remove endogenous receptors and MHCII to avoid graft-versus-host and allorecognition, respectively (73).

Future studies in larger animal species, such as dogs with hemophilia (74, 75), are planned as a step toward translation in human clinical studies.

## Author Contributions

DS is solely responsible for the content of this article.

## Conflict of Interest Statement

The author declares that the research was conducted in the absence of any commercial or financial relationships that could be construed as a potential conflict of interest.
